# ASCL1 dysfunction contributes to the pathogenesis of schizophrenia by regulating genes associated with neuronal signature formation and neuroplasticity

**DOI:** 10.1192/j.eurpsy.2024.697

**Published:** 2024-08-27

**Authors:** D. Abashkin, E. Marilovtseva, D. Karpov, V. Golimbet

**Affiliations:** ^1^Clinical Genetics Laboratoty, Mental Health Research Center, Moscow, Russian Federation

## Abstract

**Introduction:**

ASCL1 (Achaete-scute homolog 1) is a neuron-specific transcription factor involved in CNS maturation in the mammalian brain. It has been shown to be associated with schizophrenia (SZ), Parkinson’s disease, and the development of brain tumors. ASCL1 is expressed in the neuroblastoma cell line SH-SY5Y, which is a widely used model for the study of neurodevelopmental diseases, including SZ.

**Objectives:**

The aim of this work was to study the effect of functional *ASCL1* knockout on the transcriptional landscape of SH-SY5Y cells in undifferentiated and neuron-like phenotypes.

**Methods:**

For ASCL1 deletion, SH-SY5Y was sequentially transduced with two lentiviral vectors. One pLV-rtTA-Cas9-(nls)-pCMV-eGFP-PuroR-T2A-rTetR (derived from pCW-Cas9 and pEGFP-Puro) construct encoded Cas9. Stably transduced lines were selected for 3-5 days on puromycin (2 g/L). The inducibility of Cas9 expression was checked after adding the inducer oxytetracycline to the culture medium. The second construct (based on pLK05-tagRFP) encoded, a pair of guide RNAs targeting the start and end of the ASCL1 gene. The sgRNA construct was transduced into the SH-SY5Y-Cas9 cell line in parallel with a nontemplate control (NTC gRNA) as a negative control. Cas9 expression was induced with oxytetracycline for 2 days. Individual clones were obtained by serial dilutions. *ASCL1* partial deletion in the clones was confirmed by PCR followed by Sanger sequencing. Disruption of ASCL1 protein synthesis was confirmed by western blot analysis. SH-SY5Y differentiation was induced by retinoic acid (RA). The transcriptomes of mutant clones and NTC controls before and after RA-induced differentiation were sequenced using Illumina technology.

**Results:**

RNAseq data show that a wide range of genes are differentially expressed between control NTC gRNA and wild-type SH-SY5Y. This can be explained by insertional mutagenesis of lentiviral vectors and/or cellular response to the presence of lentiviral constructs. Therefore, we compared the transcriptomes of the ASCL1-del line with NTC control. Differentially expressed genes (DEGs) are predominantly associated with the pathogenesis of SZ, bipolar and depressive disorders. DEGs in ASCL1-del are involved in cell mitosis, neuronal projection, neuropeptide signaling, and formation of intercellular contacts including the synapse. During RA-induced differentiation, ASCL1 activity is restricted to the regulation of a small subset of genes involved in neuroplasticity.

**Conclusions:**

We have established a valid cellular model to study ASCL1-mediated mechanisms associated with SZ. ASCL1 dysfunction promotes SZ development predominantly before neuronal differentiation begins, slowing cell proliferation and preventing the formation of neuronal signatures.

**Disclosure of Interest:**

None Declared

